# Expression of the Extracellular Sulfatase *SULF2* Affects Survival of Head and Neck Squamous Cell Carcinoma Patients

**DOI:** 10.3389/fonc.2020.582827

**Published:** 2021-01-08

**Authors:** Yang Yang, Jaeil Ahn, Rekha Raghunathan, Bhaskar V. Kallakury, Bruce Davidson, Zuzana Brnakova Kennedy, Joseph Zaia, Radoslav Goldman

**Affiliations:** ^1^ Department of Biochemistry and Molecular & Cellular Biology, Georgetown University, Washington, DC, United States; ^2^ Department of Biostatistics, Bioinformatics, and Biomathematics, Georgetown University, Washington, DC, United States; ^3^ Department of Biochemistry, Center for Biomedical Mass Spectrometry, Boston University School of Medicine, Boston, MA, United States; ^4^ Department of Pathology, Lombardi Comprehensive Cancer Center, Georgetown University, Washington, DC, United States; ^5^ Department of Otolaryngology-Head and Neck Surgery, Medstar Georgetown University Hospital, Washington, DC, United States; ^6^ Department of Oncology and Clinical and Translational Glycoscience Research Center, Georgetown University, Washington, DC, United States

**Keywords:** head and neck squamous cell carcinoma, extracellular sulfatase Sulf-2, SULF2, heparan sulfate,, 6-*O*-sulfation, patient survival

## Abstract

Sulfation of heparan sulfate proteoglycans (HSPG) regulates signaling of growth factor receptors *via* specific interactions with the sulfate groups. 6-*O*-Sulfation of HSPG is an impactful modification regulated by the activities of dedicated extracellular endosulfatases. Specifically, extracellular sulfatase Sulf-2 (SULF2) removes 6-*O*-sulfate from HS chains, modulates affinity of carrier HSPG to their ligands, and thereby influences activity of the downstream signaling pathway. In this study, we explored the effect of SULF2 expression on HSPG sulfation and its relationship to clinical outcomes of patients with head and neck squamous cell carcinoma (HNSCC). We found a significant overexpression of SULF2 in HNSCC tumor tissues which differs by tumor location and etiology. Expression of SULF2 mRNA in tumors associated with human papillomavirus (HPV) infection was two-fold lower than in tumors associated with a history of tobacco and alcohol consumption. High SULF2 mRNA expression is significantly correlated with poor progression-free interval and overall survival of patients (n = 499). Among all HS-related enzymes, SULF2 expression had the highest hazard ratio in overall survival after adjusting for clinical characteristics. SULF2 protein expression (n = 124), determined by immunohistochemical analysis, showed a similar trend. The content of 6-*O*-sulfated HSPG, measured by staining with the HS3A8 antibody, was higher in adjacent mucosa compared to tumor tissue but revealed no difference based on SULF2 staining. LC-MS/MS analysis showed low abundance of N-sulfation and O-sulfation in HS but no significant difference between SULF2-positive and SULF2-negative tumors. Levels of enzymes modifying 6-*O*-sulfation, measured by RT-qPCR in HNSCC tumor tissues, suggest that HSPG sulfation is carried out by the co-regulated activities of multiple genes. Imbalance of the HS modifying enzymes in HNSCC tumors modifies the overall sulfation pattern, but the alteration of 6-*O*-sulfate is likely non-uniform and occurs in specific domains of the HS chains. These findings demonstrate that SULF2 expression correlates with survival of HNSCC patients and could potentially serve as a prognostic factor or target of therapeutic interventions.

## Introduction

Heparan sulfate proteoglycans (HSPGs) represent a family of proteoglycans (PG) composed of a core protein modified by the heparan sulfate (HS) glycosaminoglycan (GAG) chains (1). HSPGs direct embryogenesis, organogenesis, and physiology of nearly all adult organs from digestive, musculoskeletal, nervous to circulatory or the immune systems (2). These HS chains are linear polysaccharides composed of alternating disaccharide units of *N*-acetyl-glucosamine (GlcNAc) and D-glucuronic/L-iduronic acids (GlcA/IdoA) further modified by *N*-deacetylation and sulfation, epimerization, and variable *O*-sulfation (1). Four different sulfation sites were identified in the HS chains at the *N*-, 3-*O*-, and 6-*O*-positions of glucosamine and at the 2-*O*-position of glucuronic acid (1). Sulfation accumulates unevenly along the HS chains which generates defined HS domains of highly, partially, and non-sulfated regions (3). Sulfation editing enzymes, including the extracellular 6-*O*-endosulfatases SULF1 and SULF2, contribute to the diversity of the sulfation patterns of the HS chains which provide specific recognition sites for selective binding of protein ligands. In this way, HSPG regulates the activity of diverse signaling pathways orchestrating cell–cell and cell–matrix interactions in organogenesis (2, 4).

The known HSPG ligands include growth factors and morphogens, cytokines, chemokines, enzymes, and cell–matrix proteins which define their ability to direct the complex developmental processes (2, 5). Specificity of the interactions depends on the fine structure, especially the sulfation patterns, of the unevenly distributed HSPG domains which make them critical determinants of HSPG functions. Among the sulfation determinants, 6-*O*-sulfation is of great importance because it is essential for HS-binding of ligands including VEGF, FGF-1, FGF-10, IL8, hepatocyte growth factor, lipoprotein lipase, herpes simplex glycoprotein c, noggin, or L- and P- selectins (1, 6).

SULF2 is a neutral pH extracellular endosulfatase that specifically removes the 6-*O*-sulfate from the heparan sulfate (HS) chains of HSPG (7). SULF2 appears to be functionally redundant because SULF1 has overlapping enzymatic activities and substrate specificity based on current knowledge and kinetic studies (8). It is, however, expected that as yet unidentified differences distinguish their activities *in vivo*. The post-biosynthetic editing of the 6-*O*-sulfation pattern is a recognized regulatory mechanism strongly affecting the HSPG functions. The highly specific endoglucosamine-6-sulfatase activities of SULFs liberate the HS-binding proteins, including VEGF, FGF, or Wnt, from sequestration on the HSPG which regulates their access to their cognate receptors (6, 9, 10). Interpretation of the observations should be done with caution as contextual experimental details may differ from *in vivo* biology, but they cumulatively document the importance of SULF2 in human pathophysiology.

Head and neck squamous cell carcinoma (HNSCC), the sixth leading cancer worldwide, arises from the transformation of squamous epithelia of the oral cavity, laryngeal, and hypopharyngeal regions. The five-year overall survival rate of HNSCC patients is approximately 40% and poor outcomes are, at least in part, due to the lack of suitable markers for the detection of early stage cancers with favorable clinical outcomes. Dysregulation of HSPG is frequently observed in cancer diseases, but we have insufficient knowledge of the function of HSPG and, in particular, SULF2 in the initiation and progression of HNSCC. We have shown previously that HNSCC tumors express higher amounts of the SULF2 protein (11) and other studies documented that the distribution of SULF2 in several cancer diseases (non-small cell lung cancer, esophageal cancer, hepatocellular carcinoma or breast cancer) is associated with poor survival (10). Here we explore the effect of SULF2 expression on the HSPG sulfation in HNSCC tumors and its association with clinical outcomes.

## Materials and Methods

### Differential Expression and Survival Analysis of RNA-seq Data From the Cancer Genome Atlas

Gene expression data of 499 HNSCC patients quantified by RNA-seq in The Cancer Genome Atlas (TCGA) and corresponding clinical information were downloaded from UCSC-Xena (version: 09-14-2017) and supplemented by the TCGA group paper (12). HTseq-count values were log2(counts+1) transformed for statistical analysis. Among the 499 cancer patients, 43 patients with RNA-seq data available for both tumor and adjacent benign tissues were selected for the analysis of differential gene expression. To reduce tissue heterogeneity, a subset of 16 pairs of samples classified as squamous histology and containing at least 30% squamous epithelium was further selected from the group of 43 paired samples (12). Differential expression was analyzed by paired t-test and empirical Bayes approach was followed to validate the statistical significance. Genes that passed all analyses (p-value <0.05) were considered consistently differentially expressed between tumor and the adjacent normal tissues. In addition, gene expressions were quantified by median of RNA-seq counts value of tumor and benign tissues from the 43-paired samples from TCGA. Log transformed fold-changes (log2FC) in tumor compared to benign tissues were computed by difference of log2(counts+1) of RNA-seq data.

Evaluation of the relationship between gene expressions and clinical characteristics was performed in all 499 patients using RNA-seq data from tumor tissues. ANOVA was used to assess association between each gene expression and clinical variables with more than two attributes (e.g. tumor location) with Bonferroni adjustment for multiple comparisons. For time-to-event endpoints, we used overall survival (OS) and progression-free interval (PFI); OS was defined as the period from the date of diagnosis until the date of death from any cause or until the date of last contact and PFI was defined as the period from the date of diagnosis until the date of the first occurrence of a new tumor event such as local, regional or distant-recurrence, death attributed to cancer or the development of a new primary tumor (13). The impact of SULFs gene expression on OS and PFI was assessed using the Kaplan–Meier method where a cutoff value to define high and low expression of SULF1 or SULF2 mRNA was determined by a value that yields the smallest p-value when low vs high expression groups are compared, respectively. Multivariable adjusted Cox proportional hazards models were conducted, adjusting for age, gender, smoking status, tumor stage, and radiation therapy. To avoid loss of power, we included key confounding variables such as tumor stage and radiation therapy with <20% missing values by treating missing data as a ‘Not available’ category. Other confounding variables such as HPV status or tumor location were not included because HPV has >32% missing data and tumor locations were not statistically significant in the Kaplan–Meier analysis. Patients with tumor diagnosed as stage I and stage II are categorized as early stage while stages III and IV as late stage. Detailed patient characteristics are illustrated in **Supplementary Table 1**.

### Survival Analysis Based on IHC Staining of SULF2 Protein in Tumor Tissues

A set of 124 patients who underwent surgery for oral squamous cell carcinoma (OSCC) at Medstar Georgetown University Hospital (MGUH) between 1996 and 2014 were enrolled in the survival study in line with informed consent and protocols approved by the Institutional Review Board. Clinical data for all participants were extracted from medical records. Tumor stage was classified according to the 7^th^ Edition of the American Joint Committee on Cancer Staging system. Sections of the FFPE tumor and adjacent normal tissues were stained with an anti-SULF2 monoclonal antibody 8G1 and evaluated by a clinical pathologist as described previously (11). A combined score of intensity and distribution (both scores range from 0 to 3) was used to evaluate patient survival using both univariate analysis and models as described above. Patient characteristics are illustrated in **Table 1**. The 8G1 antibody was provided by Dr. Lemjabbar-Alaoui, University of California, San Francisco (14). Specificity of the 8G1 antibody to SULF2 was validated by Millipore (https://www.emdmillipore.com/US/en/product/Anti-Sulf-2-Antibody-clone-8G1,MM_NF-MABC586#anchor_COA). Optimization of the immunohistochemical use of the 8G1 antibody, including antigen retrieval and dilutions of the antibodies, was carried out by standard immunohistochemical procedures at the Histopathology and Tissue Shared Resource, Lombardi Comprehensive Cancer Center, Georgetown University Medical Center. Specificity of the 8G1 antibody to SULF2 in immunohistochemical staining was tested in HEK293 cells (negative control), and HEK293 cells transfected with SULF2 (positive control) embedded in FFPE blocks. Final optimization of the antibody was done using a combination of lung cancer and head and neck squamous cell carcinoma tissue sections that were positive and negative for h-Sulf2 and minus primary negative controls.

**Table 1:** Clinical information of 124 OSCC patients enrolled in the study at GUMC.

Clinical information of 124 patients from GUMC		
**Gender**	Male	68 (54.8%)
	Female	56 (45.2%)
**Tumor stage**	Stage I & II	61 (49.2%)
	Stages III & IV	61 (49.2%)
	Unknown	2 (1.6%)
**Radiation Therapy**	Yes	56 (45.2%)
	No	52 (41.9%)
	Unknown	16 (12.9%)

### IHC Staining of 6-*O*-Sulfate of Heparan Sulfate Proteoglycan and Ki67 in HNSCC Patients

Tumor and adjacent benign mucosal tissues of a subset of 40 HNSCC patients stained for SULF2 were used for paired analysis of 6-*O*-sulfated HSPG using the phage display antibody HS3A8 as described previously (15). The HS3A8 antibody was provided by Dr. Van Kuppevelt, University of Nijmegen, The Netherlands. The Ki67 antigen was stained with anti-Ki67 monoclonal antibody (Thermo Scientific) and visualized using EnVision+ horseradish peroxidase labeled polymer (Dako) according to the manufacturer’s instructions in the same sample set to evaluate the tumor cell proliferation. Briefly, 5 µm sections from FFPE tissues were deparaffinized with xylenes and rehydrated through a graded alcohol series. Slides were treated with 3% hydrogen peroxide and 10% normal goat serum for 10 min each and exposed to primary antibodies for Ki67 (Thermo Scientific) overnight at room temperature. Slides were incubated with EnVision+ HRP labeled polymer for 30 min and DAB chromagen (Dako) for 5 min. Slides were counterstained with Hematoxylin (Fisher, Harris Modified Hematoxylin), blued in 1% ammonium hydroxide, dehydrated, and mounted with Acrymount. Ki-67 was scored as proportional number of Ki-67 stained cells (in %) and intensity score in tumor tissue.

### LC-MS/MS Analysis of Heparan Sulfate Proteoglycan Sulfation in Tumor Tissues

Samples of 21 patients were selected for subgroup analysis based on SULF2 staining (10 SULF2-positive samples and 11 SULF2-negative samples). SULF2-positive tumors were defined as combined score ≥5 while SULF2-negative tumors were defined as combined score =0. Three consecutive 10 μm FFPE sections were used as technical replicates for the LC-MS/MS evaluation of heparan sulfation. An area of 2 mm diameter with homogenous tumor cell content and uniform SULF2 staining was marked by the pathologist for on-slide serial enzymatic digestion and extraction of the heparan sulfate chains as described (16). Briefly, the FFPE sections were first deparaffinized and washed with a series of xylene (Millipore) and ethanol washes (Fisher Scientific), followed by on-slide enzymatic digestion with multiple glycosidases as described (16), to cleave the polysaccharide chains into disaccharide units. The extracted heparan sulfate disaccharide units were desalted by size exclusion chromatography and analyzed by LC-MS/MS on an Orbitrap-XL as described. The relative abundances of HS disaccharides represent the composition of the HS polysaccharide chains. The mean values of the three replicates were used for further statistical analysis.

### qRT-PCR of Heparan Sulfate Biosynthetic Enzymes

Enzymes involved in the formation of 6-*O*-sulfate of HSPG including EXT1, EXT2, NDST1, HS6ST1, HS6ST2, SULF1, and SULF2 were measured by qRT-PCR. Primers of the target genes were optimized for short template sequence considering the mRNA extraction from FFPE samples. The mRNA was extracted from homogenous areas of 21 tumor samples selected by pathological examination as described above (same tissues were used for LC-MS/MS analysis of the heparan sulfate) using Pinpoint Slide RNA Isolation System II (Zymo Research). The mRNA (5 ng/μl) was used for reverse transcription (total volume 20 μl) using Superscript IV First Strand Synthesis System (Invitrogen), and 1 μl of the cDNA product was used in qRT-PCR with 100 nM primers using Power SYBR Green PCR Master Mix (Applied Biosystems). Comparative Ct method (ΔΔCT) for relative quantitation of gene expression was normalized to the expression of 18s RNA.

## Results

### Expression of SULF1 and SULF2 mRNA in Tumors Is Associated With Clinical Characteristics of HNSCC Patients

**Figure 1:**
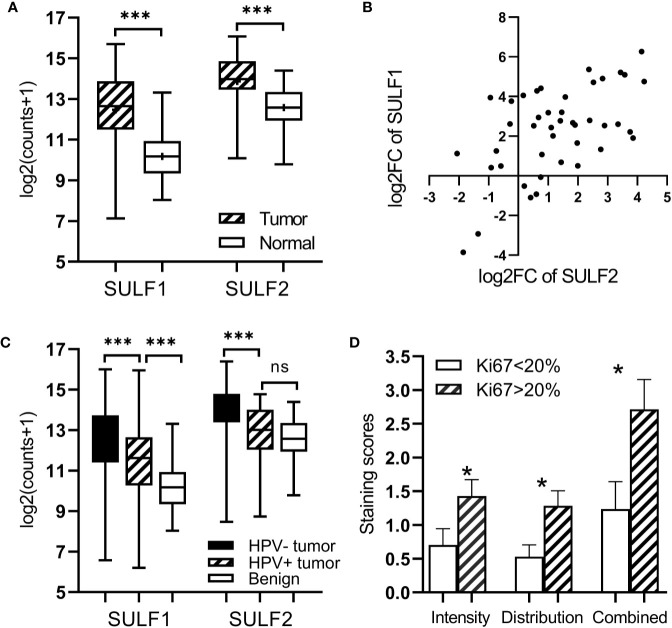
Expression of SULF1 and SULF2 in HNSCC tumor and adjacent tissues: **(A)** Upregulated SULF1 and SULF2 mRNA in tumor compared to benign tissues in patients from TCGA (p < 0.001 for both SULFs, n = 43, paired t-test). **(B)** Correlation of fold change between tumor and benign tissues for SULF1 and SULF2 in patients from TCGA (r = 0.563, p < 0.001, n = 43, Pearson correlation). **(C)** SULF1 and SULF2 mRNA in tumors from the TCGA study vary by HPV status (p < 0.001 for both, n = 335, one-way ANOVA). **(D)** IHC staining of SULF2 protein in tumor varies by percent of Ki67 IHC staining FFPE tumor sections (n = 40). * is 0.01<p<0.05, *** is p<0.001.

We analyzed differential expression of both SULF1 and SULF2 in a group of 43 patients from the TCGA-HNSC with RNA-seq results of paired tumor and adjacent benign mucosal tissue. We observed that both SULF1 and SULF2 are significantly upregulated in HNSCC tumor with 5.1-fold and 2.2-fold increase compared to adjacent benign tissues, respectively (both p < 0.001, paired t-test, **Figure 1A**). The fold-change of SULF1 and SULF2 upregulation in tumor among these 43 patients are moderately correlated (Pearson’s r = 0.563, p < 0.001, **Figure 1B**), as is the mRNA expression in tumor tissues in all 499 patients (r = 0.390, p < 0.001, **Supplementary Figure 1A**).

The TCGA cohort of 499 HNSCC patient consists of specimens from the oral cavity (n = 330, 66%), larynx (n = 113, 22%), oropharynx (n = 48, 10%), and hypopharynx sites (n = 8, 2%) (**Supplementary Table 1**). Both SULF1 and SULF2 expression in tumor samples showed significant differences among the four locations (p = 0.018 for SULF1, p < 0.001 for SULF2, one-way ANOVA, **Table 2**). SULF1 has the highest expression in laryngeal tumors which are significantly higher than in oropharynx (p = 0.028). SULF2 mRNA has highest expression in oral cavity tumors which is significantly higher than the expression in the larynx (p = 0.006) and in the oropharynx (p < 0.001). Neither SULF1 nor SULF2 differs between early and late stage tumors (**Supplementary Figure 1B**).

**Table 2:** Evaluation of the association between SULF mRNA expression in tumor tissues and clinical characteristics of 499 HNSCC patients enrolled in the TCGA study.

SULFs mRNA expression in TCGA-HNSC		
**Tumor location**	SULF1 mRNA	SULF2 mRNA
**Oral cavity (n = 330)**	11.92 ± 0.10	13.87 ± 0.07
**Larynx (n = 113)**	12.44 ± 0.17	13.38 ± 0.14
**Oropharynx (n = 48)**	11.54 ± 0.26	12.97 ± 0.23
**Hypopharynx (n = 8)**	12.04 ± 0.60	13.13 ± 0.44
**P-value**	0.018	<0.001
**HPV infection**	SULF1 mRNA	SULF2 mRNA
**HPV− (n = 275)**	12.46 ± 0.11	13.92 ± 0.08
**HPV+ (n = 60)**	11.49 ± 0.23	12.81 ± 0.18
**Normal (n = 43)**	10.17 ± 0.17	12.58 ± 0.16
**P-value**	<0.001	<0.001
**Tumor stage**	SULF1 mRNA	SULF2 mRNA
**Stages I & II (n = 97)**	11.90 ± 0.17	13.92 ± 0.12
**Stages III & IV (n = 341)**	12.09 ± 0.10	13.63 ± 0.08
**P-value**	0.398	0.058

SULF1 and SULF2 expression is represented by log2(counts+1) of RNA-seq data. One-way ANOVA was used to evaluate differences in the expression of the SULF mRNA between different groups of patients.

It is well established that HNSCC caused by human papilloma virus (HPV) infection, which mostly occurs in the oropharynx, differs from HNSCC caused by tobacco and alcohol. The HPV-related tumors have different molecular characteristics and better clinical outcomes. In the cohort of 499 patients from TCGA, 12% (n = 60) are characterized as HPV-positive, 55% as negative (n = 275), and 33% (n = 164) were not reported (**Supplementary Table 1**). We observed that 67% of the oropharyngeal tumors are positive for HPV infection (n = 32 of 48) compared to 6% of tumors of other locations (n = 28 of 452). SULF1 and SULF2 mRNA expression are significantly (p < 0.001 for both genes, one-way ANOVA) different between the HPV-positive tumors and other tissues (**Figure 1C**) with >2-fold lower expression in the HPV-positive tumors compared to HPV-negative tumors for both enzymes (**Table 2**). SULF1 is significantly higher (p < 0.001) in HPV-positive tumors compared to the subset of 43 available normal tissues but SULF2 does not differ between HPV-positive tumors and normal mucosa, suggesting that their expression is regulated differently. Low expression of both SULF1 and SULF2 in HPV-positive tumors likely explains lower mRNA in the oropharynx than other locations for both cases (**Table 2**), which is also consistent with protein expression of SULF2 observed in our previous study (11).

To further evaluate the association between SULF2 expression and aggressiveness of tumor cells, we performed immunohistochemical staining of Ki67, a cell proliferation marker, in 40 patient samples and we evaluated the distribution of Ki67 in tumor cells. We used staining ≥20% in tumor cells to define highly proliferative tissue in line with previous reports (17). We find that SULF2 staining is significantly higher in tumor samples with high Ki67 staining (p < 0.001, n = 40, **Figure 1D**). The results suggest that SULF2 overexpression is associated with proliferative activity of tumor cells. In summary, the results show that the expression of SULF 1 and 2 increases in tumors and is associated with important tumor characteristics including location, etiology, and proliferation.

### Association of SULF2 Expression With Survival Outcomes of HNSCC Patients

**Figure 2:**
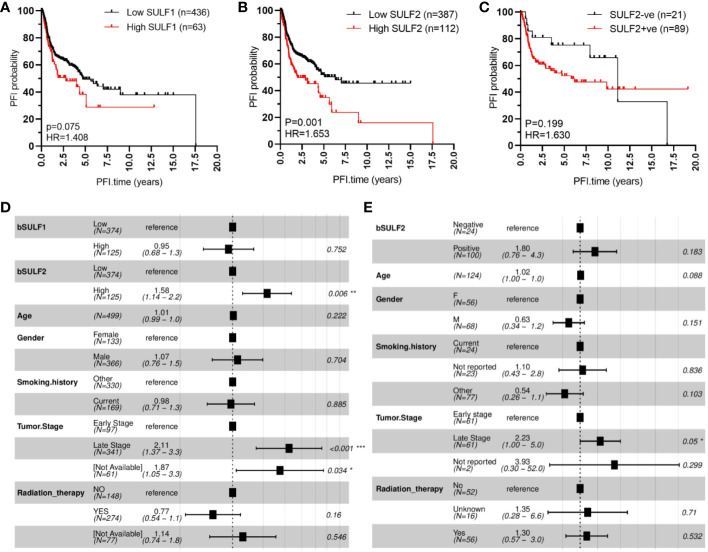
SULF expression is associated with progression-free interval (PFI) of HNSCC patients: **(A)** high SULF1 mRNA in tumor tissues is marginally associated with poor outcomes (n = 499, p = 0.075); **(B)** high SULF2 mRNA in tumor tissues is associated with poor outcomes (n = 499, p = 0.001); **(C)** positive IHC staining for SULF2 protein in tumor tissues follows a similar trend (p = 0.199). Multivariate survival model adjusted for age, gender, smoking history, tumor stage, and radiation therapy to evaluate PFI outcomes in the following context: **(D)** SULF mRNA expression in 499 patients from TCGA-HNSCC and **(E)** SULF2 protein in 124 patients from GUMC.

We evaluated first the association of SULF1 and SULF2 mRNA expression with OS and PFI in 499 patients from the TCGA-HNSC project. Univariate analysis shows that high SULF2 expression is significantly associated with poor PFI of HNSCC patients (HR = 1.653, p = 0.001, **Figure 2B**) and shows a weaker trend for SULF1 (HR = 1.408, p = 0.075, **Figure 2A**). Adjusted Cox proportional hazards model shows the expected influence of tumor stage on survival; patients with late stage tumors (stages III and IV) had significantly worse outcomes compared to patients with early stage tumors (stages I and II) (HR = 2.11, p < 0.001, **Figure 2D**). The influence of SULF2 mRNA expression remains significant after adjustment (HR = 1.58, p = 0.006) while SULF1 mRNA shows no association with the PFI of patients (**Figure 2D**).

The association was further evaluated on protein level by IHC staining of tumor FFPE sections from 124 HNSCC patients with tumor of the oral cavity (OSCC) (**Table 1**). OS and PFI for patients in this cohort are calculated according to clinical records in the same way as the TCGA analysis. We selected OSCC patients because of the more homogeneous disease and treatment characteristics and because of the dominant contribution of OSCC to the TCGA study. In our univariate model, the association of SULF2 protein with PFI of HNSCC patients shows the same trend as SULF2 mRNA; patients with tumor positive for SULF2 protein showed worse clinical outcomes than patients with negative SULF2 but the difference does not reach statistical significance (HR = 1.630, p = 0.199, **Figure 2C**) which is likely due to the small sample size and semi-quantitative nature of the IHC measurement. In the adjusted analysis, we observe significant impact of tumor stage on PFI (HR = 2.23 for late stage, p = 0.050). Impact of SULF2 protein in the adjusted model is similar to the result in the univariate model but remains insignificant (HR = 1.80, p = 0.183, **Figure 2E** **)**.

We observe similar trends in the case of overall survival with both SULF1 (HR = 1.526, p = 0.009) and SULF2 (HR = 1.482, p = 0.008) mRNA significantly associated with poor outcomes (**Supplementary Figures 2A, B** **);** SULF2 remains significant in the adjusted model (HR = 1.36, p = 0.049, **Supplementary Figure 2D**) but SULF1 does not. Tumor stage shows consistently strong influence on overall survival (HR = 2.34, p < 0.001), as expected. Patients receiving radiation therapy have significantly better survival outcomes than patients not receiving the treatment (HR = 0.51, p < 0.001, **Supplementary Figure 2D**). Similar to the impact on PFI, patients with SULF2-positive tumor tend to have worse outcomes but did not reach statistical significance (HR = 1.5, p = 0.345, **Supplementary Figures 2C, E** **)**. Overall, high expression of SULF2 mRNA is associated with both poor overall survival and progression-free interval in the HNSCC patients with a consistent but insignificant trend observed by IHC at the protein level.

### Analysis of Sulfation by IHC and LC-MS/MS

**Figure 3:**
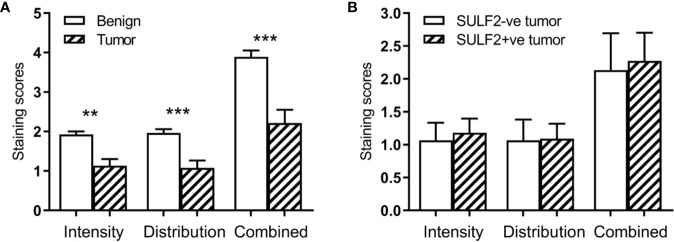
Results of IHC staining of HNSCC sections (n = 40) with an antibody to the 6-O-sulfated epitope: **(A)** Increased intensity in tumor compared to adjacent normal; **(B)** Lack of an association in the staining intensity with SULF2 status. ** is 0.001<p<0.01, *** is p<0.001.

The extracellular sulfatases SULF1 and SULF2 remove 6-*O*-sulfate groups from the glucosamine residue of the heparan sulfate chains. We analyzed the 6-*O*-sulfate content of HSPG in tumor and adjacent benign tissues by previously established IHC staining using HS3A8 antibody (15) and LC-MS/MS of disaccharide units (16) to detect whether the sulfatase activity is directly measurable in HNSCC patient tissues. The IHC staining of FFPE sections (n = 40) for the 6-*O*-sulfate (distribution, intensity and the combined score), using the HS3A8 antibody, was significantly lower (p < 0.001) in tumors compared to adjacent benign tissues (**Figure 3A**). We further expected that high expression of SULF2 protein by IHC will be associated with lower content of the 6-O sulfate in HS chains, but the analysis of the same tumor sections by SULF2 content did not show differences in 6-*O*-sulfate staining (**Figure 3B**).

**Figure 4:**
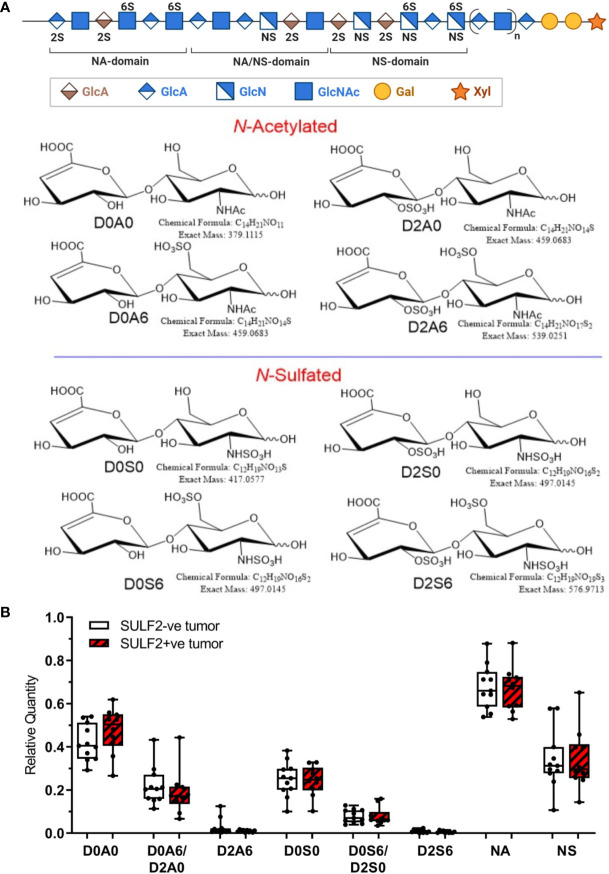
Sulfation pattern of HSPG in HNSCC tumor tissues. **(A)** Composition of HS chains represented by disaccharide units: one unsulfated disaccharide Δ^4,5^-uronic acid-*N*-acetylglucosamine (D0A0); three monosulfated disaccharides Δ^4,5^-uronic acid-*N*-acetylglucosamine-6-*O*-sulfate (D0A6), 2-*O*-sulfated Δ4,5-uronic acid-*N*-acetylglucosamine (D2A0), Δ^4,5^-uronic acid-*N*-sulfated glucosamine (D0S0); three disulfated disaccharides Δ^4,5^-uronic acid-*N*-sulfoglucosamine-6-*O*-sulfate (D0S6), 2-*O*-sulfated Δ^4,5^-uronic acid-*N*-sulfoglucosamine (D2S0), 2-*O*-sulfated Δ^4,5^-uronic acid-*N*-acetylglucosamine-6-*O*-sulfate (D2A6); one trisulfated disaccharide, 2-*O*-sulfated Δ^4,5^-uronic acid-*N*-sulfoglucosamine-6-*O*-sulfate (D2S6). **(B)** Relative quantity of the disaccharides detected in SULF2+ve tumors (n = 10) and SULF2-ve tumors (n = 11). NA represents the combined value of *N*-acetylated disaccharide units (D0A0, D0A6&D2A0, D2A6). NS represents the combined value of *N*-sulfated disaccharide units (D0S0, D0S6/D2S0, D2S6).

To further evaluate the composition of heparan sulfate chains, we analyzed composition of HS disaccharides in FFPE sections of 10 SULF2-positive (SULF2+ve) and 11 SULF2-negative (SULF2-ve) tumors from 21 HNSCC patients by LC-MS/MS (16) (**Figure 4A** **)**. However, we did not observe the expected decrease of the 6-*O*-sulfated disaccharides in the SULF2+ve tumor tissues (**Figure 4B**). D0A0 disaccharide, which represents the fraction of unmodified HS chains, is the most abundant disaccharides (40–55% of the total disaccharides in SULF2+ve tumors; 36–48% in SULF2-ve tumors). D0A0 is slightly higher in SULF2+ve tumor but this trend is not significant.

*N*-Deacetylation/*N*-sulfation enzyme reactions on GlcNAc residues are regarded as the key regulatory steps that define the polysaccharide domain structure (18). All subsequent modifications, epimerization of GlcA to iduronic acid and various sulfation reactions occur in the vicinity of the *N*-sulfated domains (18). *N*-Acetylation and *N*-sulfation, calculated by grouping corresponding disaccharide units together (D0A0, D0A6/D2A0, and D2A6 for *N*-acetylation; D0S0, D0S6/D2S0, and D2S6 for N-sulfation), showed no significant difference between SULF2+ve and SULF2-ve tumors. We observed low abundance of *N*-sulfation in the HNSCC tumors regardless of the SULF2 status (25–43% in SULF2-ve tumors, 24–44% in SULF2+ve tumors, p = 0.863, **Figure 4B**).

D0A6/D2A0 comprises most of the signal from *O*-sulfated disaccharide units in HS but reached only 16–28% in SULF2-ve tumors and 11–26% in SULF2+ve tumors, while D0S6/D2S0 showed even less abundance in both two groups (5–11% in SULF2-ve tumors, 6–10% in SULF2+ve tumors). The two pairs of isomer disaccharides D0A6/D2A0 and D0S6/D2S0 are not readily distinguishable due to their low abundance in the tumor samples. The trisulfated disaccharide unit D2S6, which is the preferred substrate of the SULF enzymes, is the least abundant (0.6% in SULF2+ve tumors; 0.9% in SULF2-ve tumors). This lower than expected abundance of *O*-sulfated disaccharides, averaged across the entire HS chain, suggests that there were too few biological specimens to discern the potential changes in D2S6 associated with SULF activities in the highly heterogenous tumor tissues.

### Expression of 6-*O*-Sulfation Editing Enzymes in HNSCC Tumor Tissues

SULF1 and SULF2 are the only two post-synthetic editing enzymes of sulfation pattern of the HSPG besides degradation of the GAG chains. However, the impact of other modifications (*e.g. N*-sulfation or 2-*O*- and 3-*O*-sulfation) or the type of protein carriers on the *in vivo* kinetic is not fully established and is expected to contribute to the differences observed in the tumor tissues. To obtain a more comprehensive view of the synthesis of the HS chains, we analyzed the expression of all 29 genes involved in the biosynthesis of heparan sulfate and HPSE, the endoglycosidase that degrades heparan sulfate chains, based on RNA-seq data and clinical information from the TCGA-HNSC (**Table 3**). We observed that 12 of the 30 genes are significantly different between tumor and adjacent normal tissues of the 43 cases with paired adjacent tissues. The same trend of differential expression of heparan sulfate genes was observed in the 16 pairs of samples that contain at least 30% squamous epithelium in the normal tissues which suggests that the analysis is not affected by tissue heterogeneity. All four genes (EXTL2, EXTL3, EXT1, and EXT2) involved in elongation of the backbone of HS chains are significantly >2-fold upregulated in tumor compared to the adjacent benign tissues. Among the 14 sulfotransferases catalyzing sulfation of the HS chains, three are downregulated in the tumor (HS3ST1, HS3ST4, and HS3ST6) while others showed no difference. Both SULF enzymes are significantly upregulated in the tumor as mentioned above. The results suggest that we should observe longer under-sulfated HS chains which is in line with the observed distribution of the disaccharides by LC-MS/MS but depends or more factors than the expression of SULF2. In addition to the differential expression analysis, we also evaluated the impact of the genes on survival outcomes and find that eight of the genes including SULF2 are significantly associated with overall survival in the adjusted model. SULF2 yields the highest HR among all the enzymes involved in this heparan sulfate synthetic pathway (**Table 3**). Unlike SULFs which specifically edit the 6-O-sulfate of HSPG, heparanase degrades the HS chains by cleaving between glucuronic acid and glucosamine of the HS chains. HPSE is reported with conflicting roles in HNSCC. Enhanced HPSE activity was previously associated with tumor invasiveness (19), but the cellular expression of heparanase in HNSCC was also associated with prolonged overall survival (20). Our analysis showed that HPSE does not differ between tumor and adjacent normal tissues (p = 0.838) and we did not observe a significant impact of HPSE on the overall survival of HNSCC patients (**Table 3**).

**Table 3:** Differential expression of enzymes synthesizing HS (n = 43 pairs, TCGA) and their impact on overall survival of patients from the TCGA study (n = 499) evaluated by the multivariate model adjusted for age, gender, smoking history, tumor stage, and radiation therapy as described in the method.

Process		Gene	Benign	Tumor	Log2FC	p-value	Hazard Ratio	p-value
Formation of the linker region	Initiate the linker	XYLT1	1711	834	−0.611	0.013	1.018	0.931
		XYLT2	1191	2650	1.180	<0.001	1.785	0.006
	Add the 2^nd^ Gal	B4GALT7	1037	1321	0.344	0.990	1.636	0.006
	Add the 3^rd^ Gal	B3GALT6	904	1257	0.688	0.287	1.290	0.181
	Add the 4^th^ GlcA	B3GAT1	19	14	−0.193	0.388	1.244	0.230
		B3GAT2	15	21	0.367	0.372	1.162	0.408
		B3GAT3	1033	2100	1.001	0.006	1.478	0.052
HS-GAG elongation	Add the first GlcNAc	EXTL2	308	614	1.018	0.001	1.405	0.062
		EXTL3	2980	5278	1.043	0.058	1.020	0.924
	Add the GlcNAc and GlcA alternatively	EXT1	2835	9371	1.745	<0.001	1.433	0.082
		EXT2	3613	6954	1.095	0.004	1.791	0.004
HS-GAG modification	N-deacetylation and N-sulfation of GlcNAc	NDST1	11591	9871	−0.149	0.622	1.260	0.199
		NDST2	98	94	−0.158	0.363	1.157	0.409
		NDST3	3	2	−0.737	0.139	NA	NA
		NDST4	1	0	0	0.871	NA	NA
	Epimerase GlcA->IdoA	GLCE	2178	1663	−0.260	0.411	1.358	0.083
	2-O-sulfation of IdoA	HS2ST1	1398	1705	0.548	0.957	1.308	0.130
	3-O-sulfation of GlcN	HS3ST1	1685	499	−1.636	<0.001	1.548	0.018
		HS3ST2	29	23	−0.549	0.255	1.088	0.636
		HS3ST3A1	284	419	0.741	0.396	1.578	0.038
		HS3ST3B1	412	427	−0.199	0.953	1.307	0.136
		HS3ST4	55	2	−2.700	0.003	NA	NA
		HS3ST5	4	4	0	0.873	NA	NA
		HS3ST6	106	31	−2.170	0.063	1.598	0.037
	6-O-sulfation of GlcNS	HS6ST1	3504	4427	0.344	0.541	1.476	0.028
		HS6ST2	696	986	0.852	0.785	1.253	0.221
		HS6ST3	7	7	0.170	0.680	NA	NA
	Removal of 6-O-sulfation	SULF1	1155	6445	2.562	<0.001	1.230	0.248
		SULF2	6108	16084	1.163	0.009	1.834	0.001
HS-GAG degradation	Cleave HS chains	HPSE	1583	1112	−0.213	0.838	1.423	0.075

Hazard ratio is calculated by comparing the risk of death for patients with high 25% gene expression to patients with low 75% gene expression.

**Figure 5:**
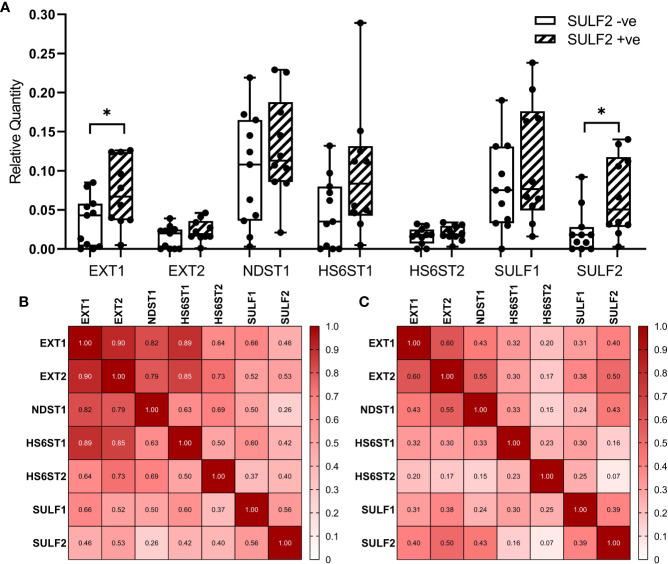
Expression of enzymes involved in the formation of 6-O-sulfate between SULF2+ve and SULF2-ve tumor. **(A)** mRNA expression of selected 6-O-S editing genes between SULF2+ve (n = 10) and SULF2-ve (n = 11) tumors extracted from FFPE sections of the 21 HNSCC patients enrolled at GUMC. EXT1 and SULF2 showed significant difference between the two groups of tumor tissues (p = 0.0391 for EXT1, p = 0.0203 for SULF2, unpaired t-test); other genes showed no difference. 18s RNA is used as endogenous control. Correlation of mRNA expression of selected 6-O-S editing genes **(B)** in 21 HNSCC patients enrolled in the study at GUMC and **(C)** in 499 HNSCC patients from the TCGA-HNSC. Numbers in each square represent the rho values of Pearson Correlation. * is 0.01<p<0.05.

We selected seven genes (EXT1, EXT2, NDST1, HS6ST1, HS6ST2, SULF1, and SULF2) from the HS-biosynthetic process, for qRT-PCR analysis of the same set of 21 FFPE sections used for the LC-MS/MS described above (**Figure 5A**). The genes cover both pre-synthetic and post-synthetic editing of 6-*O*-sulfation in the HS chains. We found that SULF2 mRNA is detectable in the tumor tissues negative by SULF2 IHC, which suggests that the production of SULF2 protein is regulated by some post-transcriptional mechanisms in addition to the mRNA expression. The EXT1 and SULF2 are significantly twofold higher in the SULF2+ve (n = 10) compared to the SULF2-ve (n = 11) tumors (p = 0.039 for EXT1, p = 0.0203 for SULF2, **Supplementary Table 2**). The other five genes are all slightly higher in SULF2+ve tumors, but the differences did not reach statistical significance. Pearson Correlation Analysis of gene expression among the 21 tumor samples revealed that EXT1 showed strong correlation to all the other enzymes (r > 0.8 for EXT2, NDST1, and HS6ST1, **Figure 5B**). Correlation between SULF2 and SULF1 mRNA is significant (r = 0.56, p = 0.008), similar to the analysis of the TCGA-HNSCC (**Supplementary Figure 1A**), and stronger than the correlation of other genes; SULF2 showed a similar correlation with EXT2 (r = 0.53, p = 0.014). The correlation of expression among the seven genes based on RNA-seq data from TCGA (n = 499) are weaker than the correlation observed in mRNA expression from FFPE tumor sections (n = 21) (**Figure 5C**), whereas the correlation between EXT2 and SULF2 remained the same (r = 0.50, p < 0.001).

## Discussion

SULF2 performs post-synthetic editing of 6-*O*-sulfation on heparan sulfate chains of HSPG which regulates binding of many ligands influencing the growth, metastasis, or immune evasion of cancers. The increased expression of SULF2 and its impact on tumor biology and/or patient survival are already established in the case of breast, liver, esophageal, or lung cancers (10). However, the function of SULF2 in HNSCC is not sufficiently explored besides our pilot IHC study which showed higher staining in the sections of HNSCC tumors (11).

In this study, we found that SULF2 overexpression in HNSCC tumor tissues is consistently observed at both mRNA and protein levels, which further confirms our previous results. SULF2 mRNA expression is significantly higher in tumor tissues that demonstrated strong staining for SULF2 protein compared to tumors negative for SULF2 staining. However, these tumors with negative SULF2 protein staining did show reliable detection of SULF2 mRNA, possibly indicating other mechanisms regulating the protein abundance besides mRNA dose on transcriptional level.

We observe a lower expression of SULF2 mRNA in HPV-positive tumors which suggests that the mechanisms of SULF2 upregulation differ in HPV-associated HNSCC. SULF1 is also elevated in tumors with even greater fold-change compared to SULF2 and shows the same trends in terms of HPV infection; however, SULF2 is more strongly associated with the clinical outcomes of HNSCC patients defined by overall survival and progression-free interval. We found that high SULF2 expression is associated with poor patient survival outcomes. In addition, we observe similar trends for the mRNA expression of SULF1. The dominant contribution of SULF2 is best seen in the adjusted models of PFI (**Figure 2D**). This suggests that the prognostic impact of SULF2 expression is independent of these clinical characteristics and is stronger than the contribution of SULF1. In addition, SULF2 protein measured by IHC follows similar expression/survival trends and is associated with higher proliferative activity of tumor cells measured by IHC staining of Ki67. Unfortunately, the analysis of SULF2 protein does not reach statistical significance due to smaller sample size (n = 124) and semiquantitative nature of the IHC staining compared to the RNA-seq gene expression study in TCGA (n = 499). We did not observe an association between SULF2 mRNA expression and tumor stage. This may be due to low representation of early stage tumors (n = 94) in the TCGA study; alternatively, SULF2 expression could be activated at an early stage of the malignant transformation process but does not further increase at later stages. These results suggest that SULF2 promotes tumor progression of HNSCC patients and deserves further attention as a potential therapeutic target in HNSCC.

To further evaluate the biological impact of SULF2 expression in HNSCC tumor, we examined the sulfation in tumor sections by HS3A8 antibodies binding preferentially the 6-*O*-sulfate (21) and by LC-MS/MS of HS disaccharides (16). We observe reduction of 6-*O*-sulfate staining and increased expressions of SULF2 in tumors, but the 6-*O*-sulfate content is not directly associated with SULF2 protein by IHC (**Figure 3B**). This could be explained by complexity of the multifactorial buildup of the epitopes. In particular, the sulfated epitopes along the HS chains are non-uniform and the average disaccharide composition across the entire HS chain may not be sufficient to resolve the local density of the sulfated HS epitope. In addition, the epitope required for antibody binding is complex and the HS3A8 preferentially binds to 6-*O*-sulfated heparan sulfate chains but other sulfate groups contribute to the interaction, like N-sulfate is as essential as the 6-*O*-sulfate for the antibody binding (21).

Our LC-MS/MS analysis of the HS disaccharides provided a more detailed view of the composition but the sulfation content is low and, as discussed above, averaged over the entire HS chain while the HS displays domain architecture with differences in local sulfate densities (22); the analysis of specific HS domains may require different analytical approaches. We therefore do not find an association between SULF2 protein staining and the trisulfated disaccharide D2S6, the preferred substrate of SULF2 *in vitro* but the least abundant fraction in our study (<1% of the disaccharide units) (15). D0A0 disaccharide, the fraction of unmodified HS chains, is most abundant and represents 36–55% of the disaccharides irrespective of the SULF2 status. *N*-Sulfation showed similar abundance of SULF2+ve (24–44%) and SULF2-ve (25–43%) tumors (p = 0.863, **Figure 4C**). The combined abundances of *O*-sulfated disaccharide units is lower in SULF2+ve tumors (18–31%) than in SULF2-ve tumors (25–38%) but the trend is not significant (p = 0.130, data not shown). Taken together, the low abundance, small number of biological specimens and high heterogeneity of tumor tissues prevent further analysis distinguishing the isomer of D0A6&D2A0 and D0S6&D2S0.

In terms of the enzymes involved in the formation of 6-*O*-sulfation, we evaluated multiple enzymes in the HS synthetic machinery in terms of mRNA expression and impact on survival. The analysis of RNA-seq data from TCGA showed that the overexpression of SULFs and their impact on the survival outcomes are higher than impact of the other enzymes involved in the process (**Table 3**). Nonetheless, several genes significantly increase in tumors and impact patient survival (XYLT2, B3GAT3, EXT2) while HS3ST1 is decreased in tumor. Further exploration of gene expression in FFPE tumor sections from HNSCC patients revealed that EXT1 is significantly elevated in SULF2-positive tumor tissues (**Supplemental Table 2**). EXT1, EXT2, and SULF2 are probably co-upregulated in tumor tissues according to the correlation analysis and higher gene expression in SULF2-positive tumors than in SULF2-negative tumors, but the mechanism underlying such upregulation still needs to be further explored. Besides SULF genes, we found that HS6ST1 showed strong correlation with EXT1 and EXT2 (r = 0.93 for EXT1, r = 0.93 for EXT2, both p-value < 0. 001) in FFPE tumor sections, but the correlations in TCGA analysis are less significant. It is plausible to speculate that reduced 6-*O*-sulfation in tumor compared to adjacent normal tissues is not resulted from the single event of SULF2 overexpression but the dysregulated biosynthetic pathway of heparan sulfate chains. Although we focus on the 6-*O*-sulfation in tumors, the overall balance of the enzymes and HS carriers will shape the structure of the HSPG domains and deserves further attention as suggested in recent studies of the functionally active “GAGosome” (23).

In conclusion, our study shows that SULF1 and SULF2 are both upregulated in HNSCC tumors at the mRNA level. SULF2 has the strongest association with PFI and OS among the HS synthesis genes. We did not observe a decrease in sulfation of the HS in the SULF2-positive tissues, but we observe reduced staining of the 6-*O*-sulfate recognizing HS3A8 antibody in the HNSCC tumors compared to normal mucosa overall. Several other genes synthesizing the HS chains are upregulated in tumor tissues, and groups of the genes (*e.g.* EXT1, EXT2, and SULF2) may be jointly regulated in the tumor tissues. We did not fully elucidate the mechanisms of SULF2 overexpression in the HNSCC tumors and its potential regulatory impact on HS-dependent signaling activities. However, we expect that the non-uniform distribution of the sulfated domains along the HS chains leads to differential binding of HS ligands in the context of HNSCC tumors and contributes to the determination of the disease outcomes.

## Data Availability Statement

The datasets presented in this study can be found in online repositories. The names of the repository/repositories and accession number(s) can be found below: https://portal.gdc.cancer.gov/projects/TCGA-HNSC, The Cancer Genome Atlas.

## Ethics Statement

The studies involving human participants were reviewed and approved by the Georgetown University-MedStar Health Institutional Review Board. The patients/participants provided their written informed consent to participate in this study.

## Author Contributions

YY analyzed the mRNA expression, carried out the data analyses, and wrote the manuscript. JA designed the statistical analysis methods and edited the manuscript. RR and JZ carried out the MS analysis of HS disaccharides. BK, ZB, and YY evaluated the IHC results. BD assisted with the design of the study, recruitment of patients, and editing of the manuscript. RG designed the study, assisted with the data interpretation, and edited the manuscript. All authors contributed to the article and approved the submitted version.

## Funding

This work was supported by the National Institutes of Health grants R21DE025732, S10OD023557, and R01 CA238455 (to RG) and CCSG Grant P30 CA51008 (to Lombardi Comprehensive Cancer Center) supporting the Histopathology and Tissue Shared Resource.

## Conflict of Interest

The authors declare that the research was conducted in the absence of any commercial or financial relationships that could be construed as a potential conflict of interest.
