# Supplementary material for: Expression of the Extracellular Sulfatase SULF2 Affects Survival of Head and Neck Squamous Cell Carcinoma Patients
Source: Front Oncol. 2021 Jan 8;10:582827. doi: 10.3389/fonc.2020.582827 (PMC7873738; doi:10.3389/fonc.2020.582827)
Supplement: Supplementary file 1 [file Presentation_1.pptx]

## Slide 1
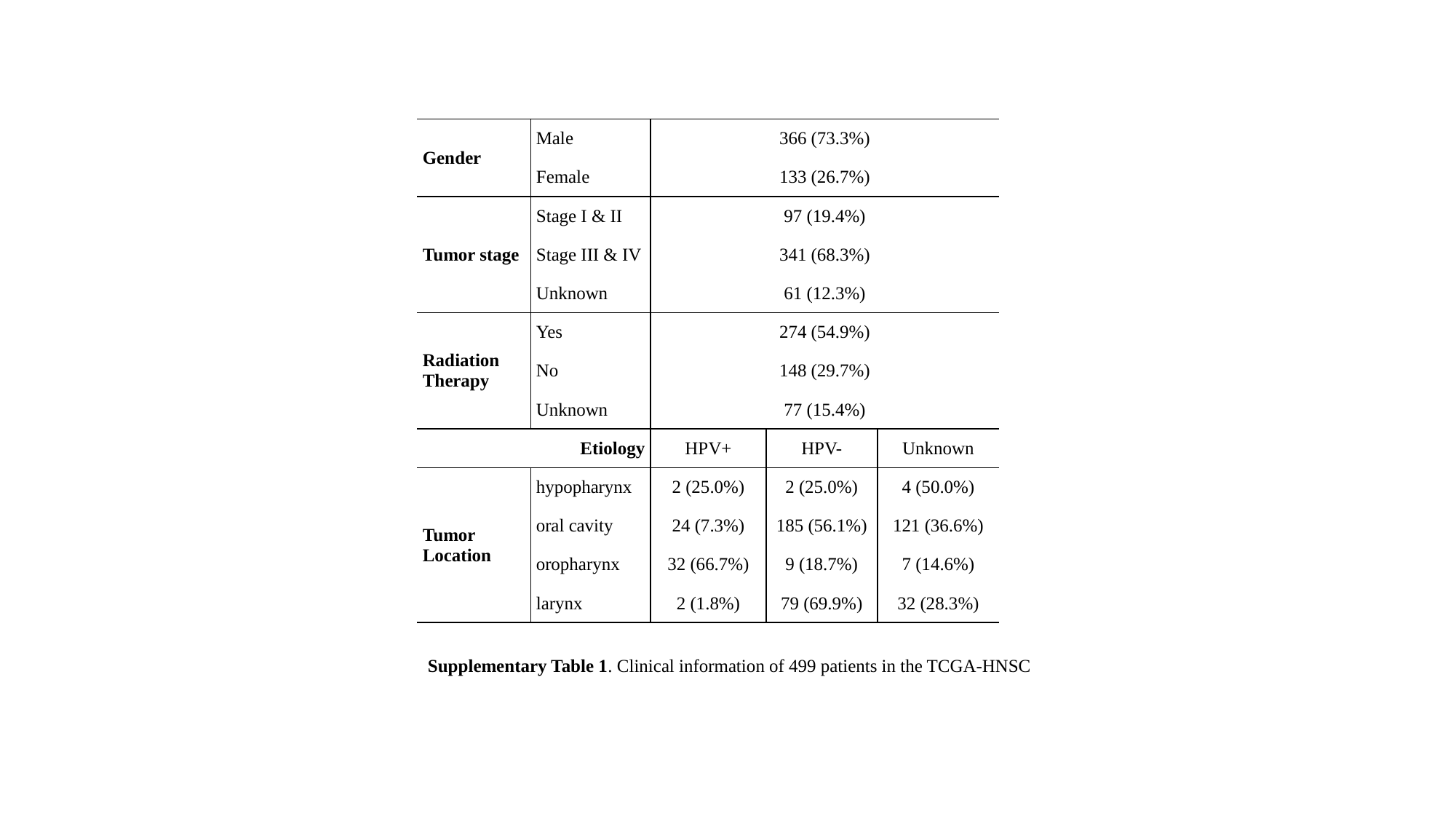

| | | | | |
| --- | --- | --- | --- | --- |
| Gender | Male | 366 (73.3%) | | |
| | Female | 133 (26.7%) | | |
| Tumor stage | Stage I & II | 97 (19.4%) | | |
| | Stage III & IV | 341 (68.3%) | | |
| | Unknown | 61 (12.3%) | | |
| Radiation Therapy | Yes | 274 (54.9%) | | |
| | No | 148 (29.7%) | | |
| | Unknown | 77 (15.4%) | | |
| Etiology | | HPV+ | HPV- | Unknown |
| Tumor Location | hypopharynx | 2 (25.0%) | 2 (25.0%) | 4 (50.0%) |
| | oral cavity | 24 (7.3%) | 185 (56.1%) | 121 (36.6%) |
| | oropharynx | 32 (66.7%) | 9 (18.7%) | 7 (14.6%) |
| | larynx | 2 (1.8%) | 79 (69.9%) | 32 (28.3%) |
Supplementary Table 1. Clinical information of 499 patients in the TCGA-HNSC

## Slide 2
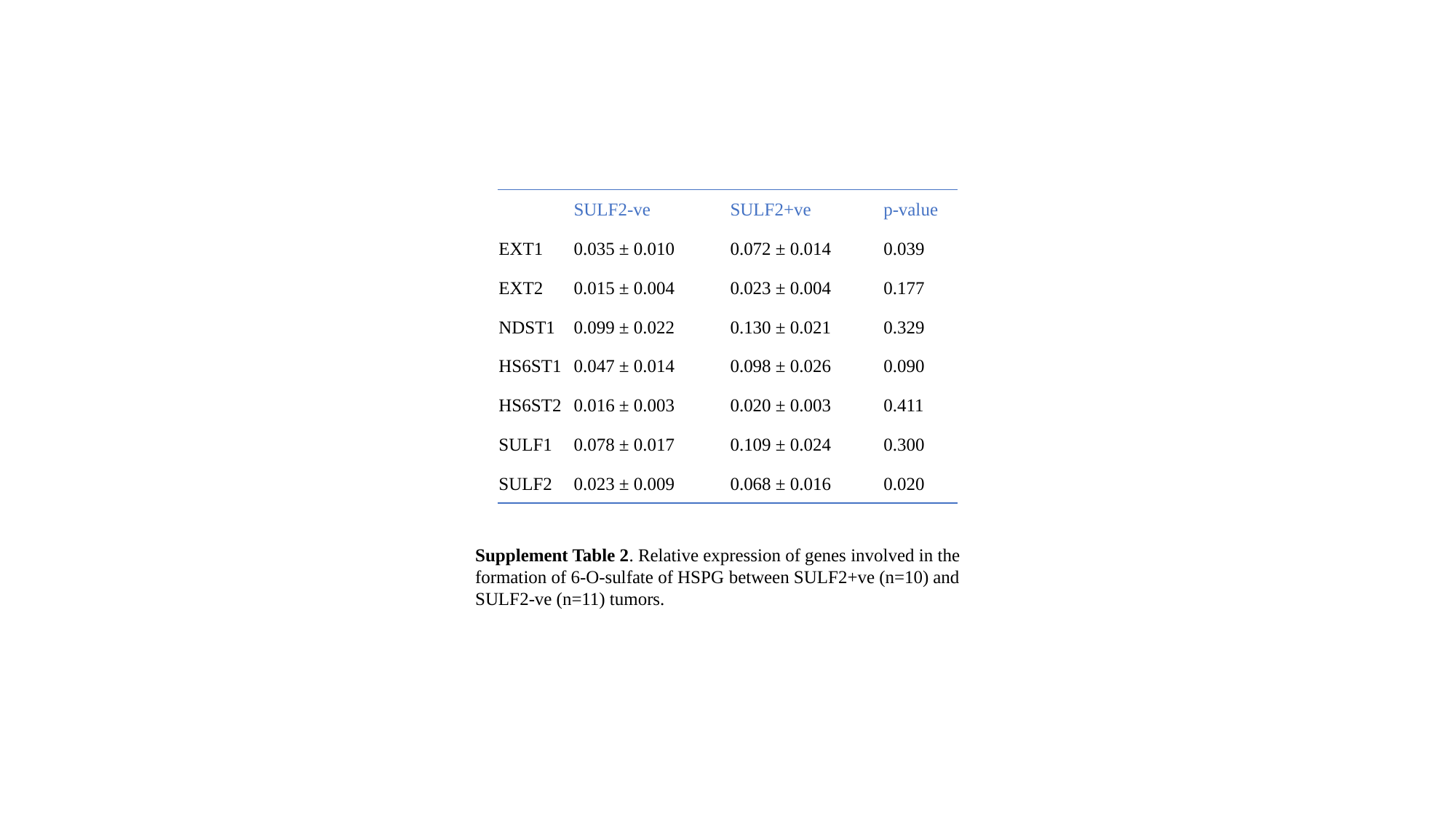

| | SULF2-ve | SULF2+ve | p-value |
| --- | --- | --- | --- |
| EXT1 | 0.035 ± 0.010 | 0.072 ± 0.014 | 0.039 |
| EXT2 | 0.015 ± 0.004 | 0.023 ± 0.004 | 0.177 |
| NDST1 | 0.099 ± 0.022 | 0.130 ± 0.021 | 0.329 |
| HS6ST1 | 0.047 ± 0.014 | 0.098 ± 0.026 | 0.090 |
| HS6ST2 | 0.016 ± 0.003 | 0.020 ± 0.003 | 0.411 |
| SULF1 | 0.078 ± 0.017 | 0.109 ± 0.024 | 0.300 |
| SULF2 | 0.023 ± 0.009 | 0.068 ± 0.016 | 0.020 |
Supplement Table 2. Relative expression of genes involved in the formation of 6-O-sulfate of HSPG between SULF2+ve (n=10) and SULF2-ve (n=11) tumors.

## Slide 3
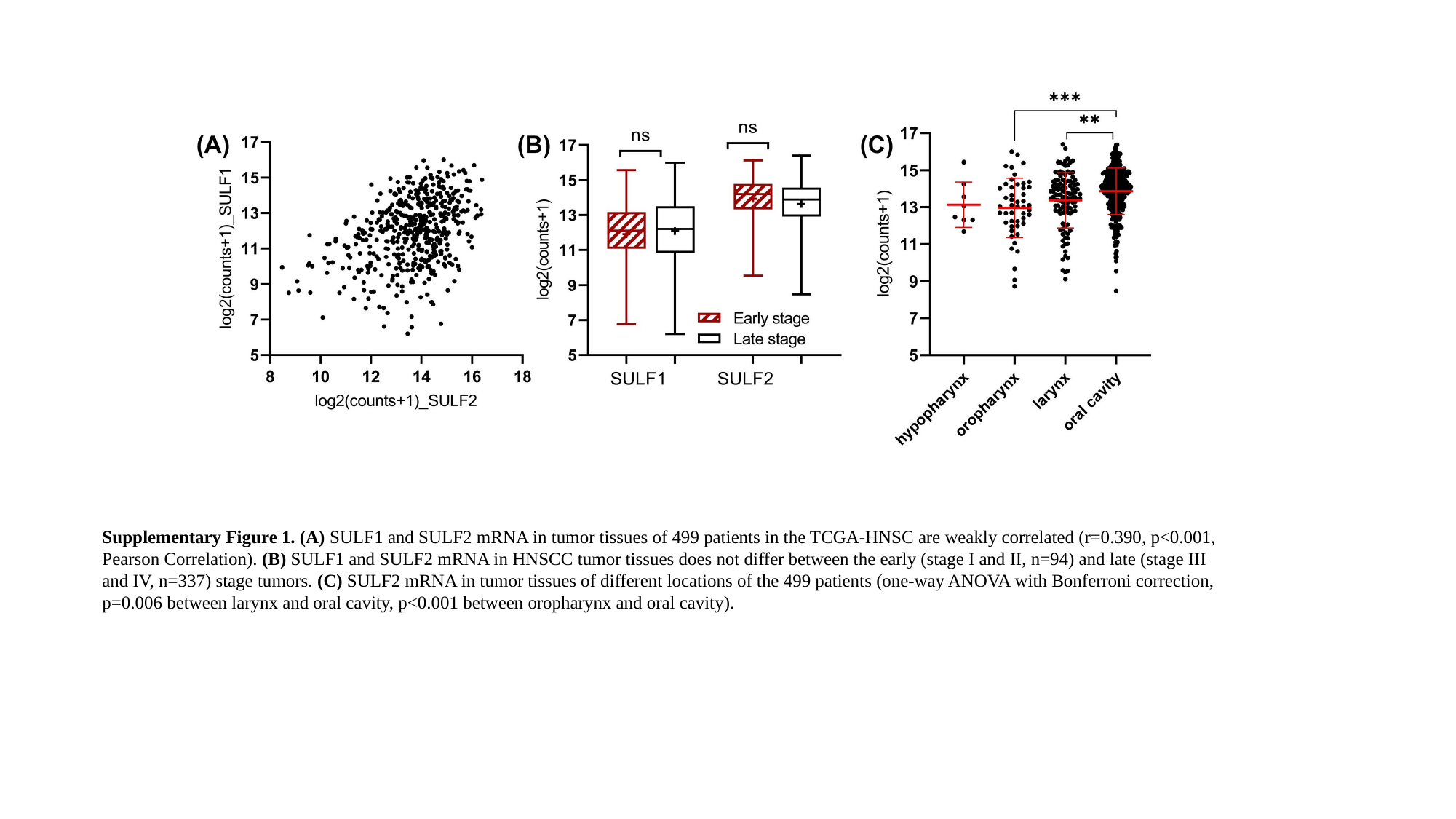

Supplementary Figure 1. (A) SULF1 and SULF2 mRNA in tumor tissues of 499 patients in the TCGA-HNSC are weakly correlated (r=0.390, p<0.001, Pearson Correlation). (B) SULF1 and SULF2 mRNA in HNSCC tumor tissues does not differ between the early (stage I and II, n=94) and late (stage III and IV, n=337) stage tumors. (C) SULF2 mRNA in tumor tissues of different locations of the 499 patients (one-way ANOVA with Bonferroni correction, p=0.006 between larynx and oral cavity, p<0.001 between oropharynx and oral cavity).

## Slide 4
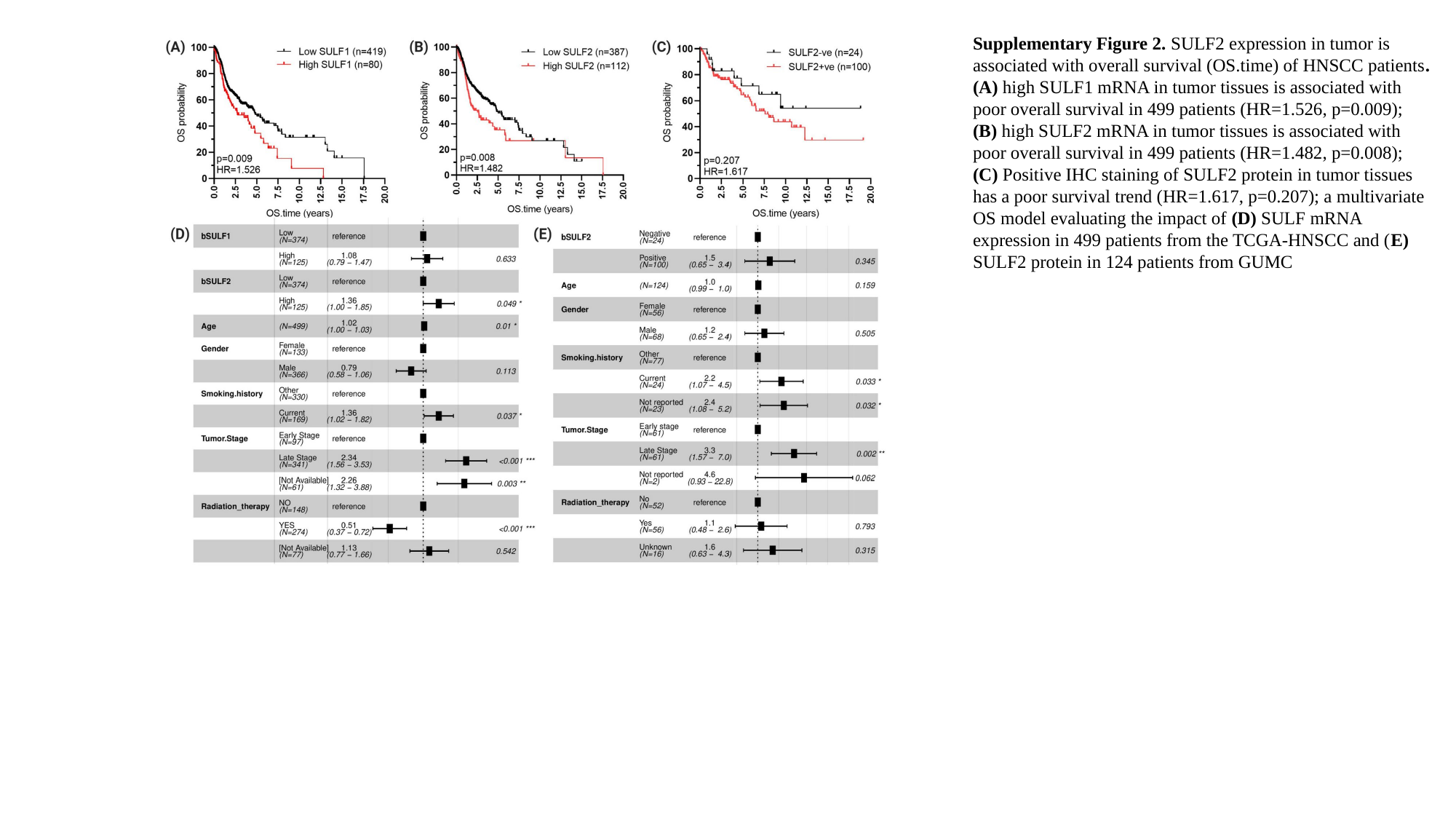

Supplementary Figure 2. SULF2 expression in tumor is associated with overall survival (OS.time) of HNSCC patients. (A) high SULF1 mRNA in tumor tissues is associated with poor overall survival in 499 patients (HR=1.526, p=0.009); (B) high SULF2 mRNA in tumor tissues is associated with poor overall survival in 499 patients (HR=1.482, p=0.008); (C) Positive IHC staining of SULF2 protein in tumor tissues has a poor survival trend (HR=1.617, p=0.207); a multivariate OS model evaluating the impact of (D) SULF mRNA expression in 499 patients from the TCGA-HNSCC and (E) SULF2 protein in 124 patients from GUMC
